# Dr. Adams, on Vaccination

**Published:** 1805-09-01

**Authors:** Joseph Adams

**Affiliations:** Berner's Street


THE
Medical and Phyfical Journal.

VOL. XIV.] SEPTEMBER 1, 1805. [NO. LXXIX.

*Printed for R. PHILLIPS, by W. Thorne, Red Lion Court, Fleet Street, London.*

### To Dr. BRADLEY.

DEAR SIR,

HAVE the goodness to permit an old friend to say a few words in his own defence, when attacked on all sides, and in all manner of ways. It is not many months since I was taken severely to task for being candid to Mr. Goldson. This I bore with becoming patience, not thinking it a crime to be civil. But only a month ago I have been accused of being uncivil to this same gentleman, or, what is still worse, of mistaking his meaning. Now, having for many years past been careful not to quarrel with any one, I little expected that those who should be the guardians of medical literature would have taken pains to foment discord.

"We apprehend," says a writer in one of the medical periodical publications, "Dr. A. has mistaken Mr. Goldson, when he makes him *say, in an unqualified manner,* that vaccination in the hand may be relied on, though it cannot in the arm. On turning to the passage in Mr. G's book, we find only that he conjectures the pustule in the hand, from having a closer resemblance to the casual vaccine pustule, may possibly be more effectual in preventing small-pox."

It is impossible not to approve of the Reviewer's industry in turning to the passage in Mr. Goldson's book, but it seems to have required much more industry to overlook the passage in my book, which, it is to be presumed, was open before him whilst *under review.* Perhaps, however, if he overlooked it, on that occasion, he may meet with it here, as his work makes occasional reference to the Medical and Physical Journal. My words are as follow:

"However, the most respectable among those who maintain this opinion, is now *led to believe* that if a person is vaccinated in the hand, he may be secure from the small-pox for life; but if in the arm, only for a time."

Mr. Goldson's words are, "*I was induced to infer.*"

My words, "*led to believe.*"

The Reviewer's words, "*to say in an unqualified manner.*"

Having thus renewed my correspondence with you, let me trouble you with a few notes, as they were collected in the Small-pox Hospital. Though I am preparing them in a better form, yet the important questions now agitated, render it the dnty of every one who has facts to produce, to be early in communicating them.

I shall begin with reminding you of an aphorism delivered by the first person who taught us to discriminate these subjects with accuracy, viz. that *every morbid poison has its distinguishing character;* and that however this may be modified on the first appearance of the local action, by any peculiarity of the constitution in the subject or atmosphere, still, in the progress of the disease, these adventitious circumstances will cease, and the true character of the poison show itself.

That keeping these objects in view, our business should be, in all chronic cases, to rest till we can ascertain the disease by its true character; and in acute cases to regulate our practice by the symptoms which show themselves. When these subside spontaneously, or by our remedies, the disease will assume its true character, and should be treated accordingly.

I have been led to these reflections by two most remarkable cases, which have lately occurred at the Small-pox Hospital. The first was a young woman about twenty-five years old, who had gone through small-pox twenty years before, and was severely enough marked with it. She applied without any introduction to the hospital, on accoun of fever, and an almost universal eruption, which she conceived to be small-pox. It was soon discovered that the progress of the disease was different from that of small-pox; but as no urgent symptoms occurred, she remained more than a week without the use of any powerful remedy. By degrees it was seen, that though some of the eruptions appeared purulent, yet the early ones became dry, the cuticle falling off, and discovering a circumscribed red spot underneath it. The fever assumed the true hectic form, and the spots, particularly about the joint of the humerus and fore arm, assumed the true venereal character. A regular course of mercurial friction removed all the symptoms, and the patient is now convalescent.

The second case was of a woman who twenty years before had been inoculated by a gentleman celebrated for his his success in that branch of the profession. Her situation was soon reported to the hospital, and the above gentleman, on the following day, applying to us for vaccine matter, was informed of the event. He was so good as to visit his patient, and examine his register. It does not appear that he made any objection to the reality of the disease, but discovered by his register, that the subject had been inoculated by his assistant, which rendered it uncertain whether the progress of the disease had been regular. The symptoms not being very urgent, and the progress of the eruption somewhat irregular, no active remedies were applied. In a very few days the true venereal character showed itself much more strikingly than in the first instance; the eruptions being fewer, somewhat larger, in solitary clusters, and situated in those parts where there was most reason to expect them.

It is most likely that at another time these cases would not have found their way to St. Pancras; but the occasional occurrence of small-pox after cow-pox, has brought forward a number of second attacks of small-pox, which tho' well authenticated, might otherwise have been concealed, or left unrecorded. This of course has taught the public to expect still more. Happily for mankind, the instances are rare after either vaccination or inoculation; but it is truly surprising that men, who are daily witnessing the various deviations from the usual course of Nature in organic structure, in the common actions of health and disease, should refuse their assent to facts so well established, and so much within the line of probability. I know it has been said, that these accounts of second small-pox have never been mentioned till the occurrence of the disease after vaccination; but this is so far from the truth, that Diemerbrock, who wrote in the middle of the seventeenth century, very circumstantially relates the history of four persons, (brothers and sisters) who had all of them the small-pox severely a second time. In this account he could not well be mistaken, as the family were inmates of his own house. His words are, "Nam erant nostri domestici, in quos singulis fere horis occuli convertebantur." His son, in his annotations on the cases, speaks of them as rare instances, but without expressing the smallest doubt of their reality. Forestus, who wrote much earlier, admits the possibility of a second recurrence.

However, instances in our own days are more to the purpose, because we have it in our power to examine their validity. These are now become so numerous, and well

 authenticated, authenticated, that I was surprised in your last number to find the case of Miss Price, which I had before related, brought as a fresh communication. However, the little addition at the close, which afforded the opportunity of a second time introducing *Mr. Travers's firebrands,* explained the cause of the insertion of the case.

If I had not had the pleasure of Mr. Travers's acquaintance more than thirty years, if I had not for that time witnessed his good intentions and the strength of his understanding, I should still have required no proofs that he was actuated by the purest motives in his eloquent Phillippic. Mr. Travers could not be aware that the small-pox is so constantly epidemic in London, that no person who has not had the disease thinks himself safe in passing a night in the metropolis. That this has been the case not only since inoculation has been introduced; but that as far back as we can trace any correct accounts, pest houses have been erected in the neighbourhood of other cities and large towns, to which those who were seized with small-pox were sent, but that even a separate hospital for small-pox in London, is of modern date. Nor was it to be expected that Mr. Travers should have made himself acquainted with the writings of the great master in this disease, of whom Boerhaave says, that every physician should read his Treatise on the Small-pox thirteen times over. Every medical man will know I am speaking of Sydenham, and also that Sydenham died before inoculation was known in Europe. Yet we find inoculation was not necessary to spread the disease *universally* through London, under certain constitutions of the atmosphere. His words, as translated by Dr. Swan, are,

"The small-pox in those years it is epidemic, when it is mild and regular, usually begins about the vernal equinox; but in those years when it is not only epidemic, but likewise irregular and of a more dangerous tendency, it sometimes appears sooner, viz. in the month of January, seizing whole families, and sparing none, of what age soever, unless they have already had it."

Now, Sir, knowing Mr. Travers well enough to be satisfied of his love of liberty, of the genuine sentiments of affection with which his heart is stored, I can have no doubt how he would conduct himself if, he saw a lovely little infant whose parents are too obstinate, if you please to use such an expression, to permit it to be vaccinated. He would, by all the eloquence he is master of, endeavour to remove their prejudices; but if he was unsuccessful, would would he coolly condemn the innocent infant to the horrors of so dreadful a disease? To this it will be answered, that by the tenderness shown to this infant, a disease may be spread over a whole neighbourhood. This answer is admissable from any but a medical practitioner of London. They know that the town is never free from the disease, and that the only preservative is an early vaccination; which it is every ones duty to inculcate.

There is one argument against inoculation for small-pox, which deserves the fairest investigation, not only on account of the source from which it is derived, but on account of the facts by which, like every thing from that source, it is supported. This is, that the comparative number of deaths by small-pox have been greater in London since the introduction of inoculation. There are, however, two reasons for this. First, that the number of children reared is greater; and it is well known that London children rarely take the small-pox at a very early age. Next, that since the introduction of inoculation, the terrors of the small-pox have gradually lessened. Before that important discovery, few were willing to trust themselves in London without having gone through the disease, and families were always on their guard not to hire servants who were liable to it. 'Tis much to be regretted that this caution is so much lessened. Almost all the females in the Small-Pox Hospital are servants from the country. Many of the men are of the same description, or labourers. It is still more surprising that we have had several soldiers from the Guards. Though I have been particular in my inquiries, I do not recollect an instance of a grown person, a native of London, who has applied for admission. The evil therefore may be remedied without difficulty. Women who migrate to London are so anxious to be settled in service, that few, if any, will refuse to be vaccinated, on condition of being hired; and this may be done with little or no interruption to their common employment.

Since the invaluable Jennerian discovery, there can be no objection to certain legal restraints on inoculation for the small-pox. It has been known from the time of Sydenham, that there are certain constitutions of the air more favourable to the extension of that disease than others. When they occur, whether inoculation is permitted or not, no caution will secure such as are still liable to the disease, and remain in town. It does not appear that such a constitution has occurred since the introduction troduction of vaccination till the present season; and even now, the mortality is considerably less than might be expected. This can only be attributed to the very large proportion of vaccinated children. The mildness of the remedy, and the laudable zeal of the friends to the new discovery, have extended the practice much further than inoculation for small-pox ever attained; and that it will become universal in a few years can scarcely be doubted. But I need not remind every medical man, that our present knowledge will by no means enable us to ascertain how long the sources of small-pox infection may remain in London after the disease has disappeared. Dr. Jenner relates the case of a boy, who was infected by remaining near the spot where the grave was opened of a person who twelve years before had died of the small-pox. Before therefore we can be secure from the disease without vaccination, it will be necessary to shut up for ever all our present church yards and burying grounds. The furniture of all our hospitals and workhouses should be destroyed, and the walls fresh plastered. The same should take place in every house, and, perhaps, every alley, in which the disease may, at any time, re-appear. These are only a few of the difficulties to be encountered; however there are none but what may be overcome, and ought to be attempted; but till this is seriously begun, the only security for an inhabitant of London must be early vaccination.

This digression has led me from the subject I began with. Since the late alarms concerning small-pox after vaccination, we have had applications of all kinds: some to be inoculated, who had been vaccinated. All these have stood the test without a single exception. Others with suspicious eruptions; but none of them have added to the catalogue of unsuccessful vaccination: and I cannot help thinking, when we reflect on the numbers vaccinated, that the very few instances of consequent small-pox, are not, if fairly estimated, greater than would have occurred after the same number of casual or inoculated small-pox.

That kind of eruption which Dr. Willan calls *favus*, and which is one of the eruptions that Dr. Jenner considers as infectious herpes, has been very common. It has affected several whole families; even the elder branches have not entirely escaped in their fingers which come in contact with their children. The principal peculiarity of it, is, that it seems to preserve no certain periods in in its progress, continuing to break out afresh repeatedly as it dries, and even in the neighbourhood of the parts last affected. It dries also with a foveolous, somewhat resembling the cell of a honeycomb in the regularity of its figure; but the cavity is extremely superficial, and never leaves a pitting. Many of these cases were called, or suspected of being, small-pox after vaccination.

The following history is remarkable for the number of persons who had previously gone through small-pox, and were inoculated by nursing the same child. It also furnishes another instance of the manner in which vaccine and variolous virus infect under different stages of either.

Charles Pigott, an infant, had the small-pox. He had two sisters; the eldest of which was removed from the house as soon as the disease was ascertained. The youngest remained at home.

They were both vaccinated fourteen days after Charles began to sicken. Nothing particular occurred in the eldest.

The insertion in the youngest appearing backward, vaccination was repeated. Before the second insertion could produce any effect, the first came forward and continued its progress. On the eleventh day from the insertion the areola was formed. Three days afterwards some variolous pustules appeared. These dried in a few days, and the vaccine pustule somewhat later than in the elder sister.

Whilst Charles was ill, he was occasionally nursed by his father, mother, a maid servant and her mother, all of whom had gone through the small-pox. The father and mother had pustules in the face, with fever and swoln glands at the neck. The maid had pustules on her arms and fever, and the maid's mother had pustules in the face.

These are only a few of the facts with which the hospital has furnished me, and which, as I find leisure, I shall occasionally offer you.

*P. S.* We have just received into the Hospital a woman, brought us by Mr. Hodges, whose children were vaccinated in Fulwood's Rents. She is said to have received the small-pox, after a regular vaccination at Wooburn, six years past. On enquiry it appears that there was no regularity in the progress of her arm, which was never examined by the surgeon who performed the operation. In your next you shall have more minute particulars.

I am, &c.
JOSEPH ADAMS.

*Berner's Street,*
*August* 20, 1805.

